# Poster Session I - A65 RETROSPECTIVE ANALYSIS OF OUTCOMES AFTER TRANSCATHETER ARTERIAL ANGIOEMBOLIZATION IN PATIENTS WITH NON-VARICEAL UPPER GASTROINTESTINAL BLEEDING

**DOI:** 10.1093/jcag/gwaf042.065

**Published:** 2026-02-13

**Authors:** M Fazal, K Labib, K Caldwell, A Hashmi, R Owen, S Zepeda Gomez

**Affiliations:** University of Alberta, Edmonton, AB, Canada; University of Alberta, Edmonton, AB, Canada; University of Alberta, Edmonton, AB, Canada; University of Alberta, Edmonton, AB, Canada; University of Alberta, Edmonton, AB, Canada; University of Alberta, Edmonton, AB, Canada

## Abstract

**Background:**

Non-variceal upper gastrointestinal bleeding (NVUGIB) is associated with significant morbidity and need for hospital admission. The mainstay of management is early medical and endoscopic assessment and treatment. However, in some cases, endoscopic intervention is unsuccessful or not feasible. Interventional Radiology guided transcatheter arterial angioembolization (TAE) or surgery is recommended in cases of re-bleeding or failed endoscopic hemostasis.

**Aims:**

Our aim was to describe the characteristics and outcomes of patients with NVUGIB who underwent TAE for bleeding control with or without previous endoscopic assessment/therapeutic intervention.

**Methods:**

Retrospective analysis of all patients with diagnosis of NVUGIB who underwent TAE for NVUGIB at the University of Alberta Hospital between January 2020 – June 2025. We analyzed demographic data, comorbidities, medications, clinical presentation, lab investigations, blood transfusion requirements, endoscopic findings and interventions, and interventional radiology findings to assess the outcomes.

**Results:**

Sixty patients who underwent TAE were identified, 42 (70%) were males and the mean age was 63 years (range 30-90). The most frequent presentation was melena (61.6%), followed by hematochezia (31.6%). Twenty-three patients (38%) were on proton pump inhibitor therapy at presentation and 14 patients (23.3%) reported current NSAID use.

Endoscopic assessment was performed in 45 (75%) patients, bleeding from peptic ulcer disease was identified in 93% of the patients followed by malignancy (6%) and vascular malformations (2%). Most of the patients (85%) required blood transfusion before endoscopy. Twenty-seven (60%) patients that ultimately went to TAE underwent some form of endoscopic intervention. Twenty-three patients (38%) underwent more than one endoscopic evaluation/intervention before being referred to Interventional Radiology. CT angiography showed active extravasation in 23 patients. Twenty-six (43%) patients who underwent TAE showed active bleeding at the time of the procedure while 34 (57%) had prophylactic embolization. Eleven patients (18%) experienced re-bleeding after embolization, nine had repeat endoscopy and 2 new TAE attempt. Five (8.3%) patients died from bleeding complications.

**Conclusions:**

Transcatheter arterial angioembolization is an effective hemostatic method and should be a second line therapy for patients with NVUGIB that experience re-bleeding after endoscopic treatment.

**Funding Agencies:**

None

